# The Use of Artificial Intelligence Algorithms in the Prognosis and Detection of Lymph Node Involvement in Head and Neck Cancer and Possible Impact in the Development of Personalized Therapeutic Strategy: A Systematic Review

**DOI:** 10.3390/jpm13121626

**Published:** 2023-11-21

**Authors:** Luca Michelutti, Alessandro Tel, Marco Zeppieri, Tamara Ius, Salvatore Sembronio, Massimo Robiony

**Affiliations:** 1 Clinic of Maxillofacial Surgery, Head-Neck and NeuroScience Department, University Hospital of Udine, p.le S. Maria della Misericordia 15, 33100 Udine, Italyalessandro.tel@asufc.sanita.fvg.it (A.T.);; 2 Department of Ophthalmology, University Hospital of Udine, Piazzale S. Maria della Misericordia 15, 33100 Udine, Italy; 3 Neurosurgery Unit, Head-Neck and NeuroScience Department, University Hospital of Udine, p.le S. Maria della Misericordia 15, 33100 Udine, Italy

**Keywords:** machine learning, deep learning, artificial intelligence, oral cancer, head and neck cancer, prognosis, therapy, follow-up, recurrence, maxillofacial surgery

## Abstract

Given the increasingly important role that the use of artificial intelligence algorithms is taking on in the medical field today (especially in oncology), the purpose of this systematic review is to analyze the main reports on such algorithms applied for the prognostic evaluation of patients with head and neck malignancies. The objective of this paper is to examine the currently available literature in the field of artificial intelligence applied to head and neck oncology, particularly in the prognostic evaluation of the patient with this kind of tumor, by means of a systematic review. The paper exposes an overview of the applications of artificial intelligence in deriving prognostic information related to the prediction of survival and recurrence and how these data may have a potential impact on the choice of therapeutic strategy, making it increasingly personalized. This systematic review was written following the PRISMA 2020 guidelines.

## 1. Introduction

Artificial intelligence in recent years has spread to all fields, from socio-economic to health care. The purpose of this systematic review is to propose an overview of the applications of artificial intelligence algorithms in oncology in head and neck cancer patients and to focus on the assessment of lymph node status through these new technological tools.

Head and neck cancer represents the sixth most common cancer in the world, with about 630,000 new patients diagnosed each year and more than 350,000 deaths each year. They are lethal cancers that have a high rate of metastasis and recurrence [1,2]. The application of artificial intelligence algorithms can potentially be one of many new tools at our disposal to better manage this disease [3,4]. There are already several recent studies that have investigated the application of artificial intelligence in the assessment of oncological outcomes, such as the study conducted by Chinnery et al. (2021) that evaluated different prognostic prediction models through the application of artificial intelligence and radiomics, demonstrating how these tools may have potential application in the clinical setting (although further studies are needed regarding the creation of standardized protocols) [5].

When we talk about artificial intelligence, we refer to a branch of computer science that deals with creating algorithms tasked with performing tasks traditionally performed by human intelligence. Machine learning (ML) is within this branch; it is a subset of artificial intelligence that allows computers to learn through data input. In addition to ML, there is also deep learning (DL), which is a subset of artificial neural networks that fall into the group of artificial intelligence and function by mimicking the functioning of the neural networks in our brains [3].

The use of artificial intelligence algorithms (in the analysis of clinical, epidemiologic, radiomic, histologic, and genomic data) in the field of oncology is proving to be a potential tool for the study of pathogenetic mechanisms, diagnosis, prediction of malignant transformation of precancerous lesions, and prognostic evaluation, both through the study of known prognostic and predictive factors and through the identification of new ones. Therefore, these computational strategies would enable improved research and prognosis of head and neck cancer [3,6,7,8].

There have been several studies in the literature that evaluate the clinical applications of these algorithms for the management of head and neck cancer. ML and AI have shown to be useful tools for grading, staging, prognostic evaluation, predicting response to therapy, and deriving information on prognostic endpoints, such as overall survival (OS), through the analysis of radiomic data. A significant and conspicuous source of data being analyzed by these machine learning and deep learning algorithms is imaging (e.g., CT, MRI, and PET images) [4,9].

Given that imaging data are an important source of information, the use of machine learning in the analysis of radiomic data can have the goal of creating models that reflect the genesis and evolution of head and neck cancer. Thanks to radiomics, quantitative features can be extracted from conventional medical images and combined with other data, such as molecular biomarkers or clinical data, to assess tumor status more accurately with positive repercussions on diagnosis and therapy, with the latter being increasingly personalized [10].

Prognostic evaluation is critical, as this has a significant impact on the choice of therapeutic strategy. The aim of this systematic review is to provide an overview of the applications of artificial intelligence algorithms in the prognostic evaluation of head and neck cancer patients; in particular, the creation of prognostic models and their impact on the therapeutic strategy. Considering that more than 65% of patients with squamous cell head and neck cancer have recurrent or metastatic disease [11], we understand how crucial it is to have at our disposal innovative tools that can best predict the patient’s prognosis and to identify those patients who present greater risk of recurrence and thus would benefit from a particular treatment compared with the standard treatment.

We also decided to focus on the evaluation and prediction of lymph node status as a prognostic factor. Lymph node metastasis is the main way of dissemination of head and neck carcinoma, and its presence has a substantial impact on the prognosis and consequent therapy [12].

## 2. Materials and Methods

This study was conducted according to the preferred reporting items for systematic review and meta-analyses (PRISMA) statement [13]. The PRISMA checklist is reported in the Supplementary Materials.

The research question of this systematic review was built according to the PICOS framework (participant, interventions, comparators, outcomes, and study design) (Table 1) and can be summarized as follows: “Does artificial intelligence algorithm-mediated prognostic evaluation of head and neck cancer patients provide useful data to improve and personalize therapeutic strategy?”.

This review was registered in the PROSPERO database (International Prospective Register of Systematic Reviews; ID number 474750).

### 2.1. Literature Search

The literature search was performed in accordance with the PICOS framework (Table 1); specifically, the items “participants” and “outcome” were used to compose the query. The query used was: ((artificial intelligence) OR (machine learning) OR (deep learning)) AND ((oral cancer) OR (head and neck cancer) OR (OSCC) OR (mouth neoplasm)). Using combinations of keywords, the literature search was conducted until 29 September 2023 by consulting the following medical literature databases: MEDLINE, Cochrane Central Register of Controlled Trials (CENTRAL), ClinicalTrials.gov, ScienceDirect, Embase, Scopus, and CINAHL.

For database searching, filters were applied to select English-only articles conducted on human species with publication dates between 2013 and 2023. The selected articles were clinical trials, randomized clinical trials, cohort studies, original articles, and research articles.

**Figure 1:**
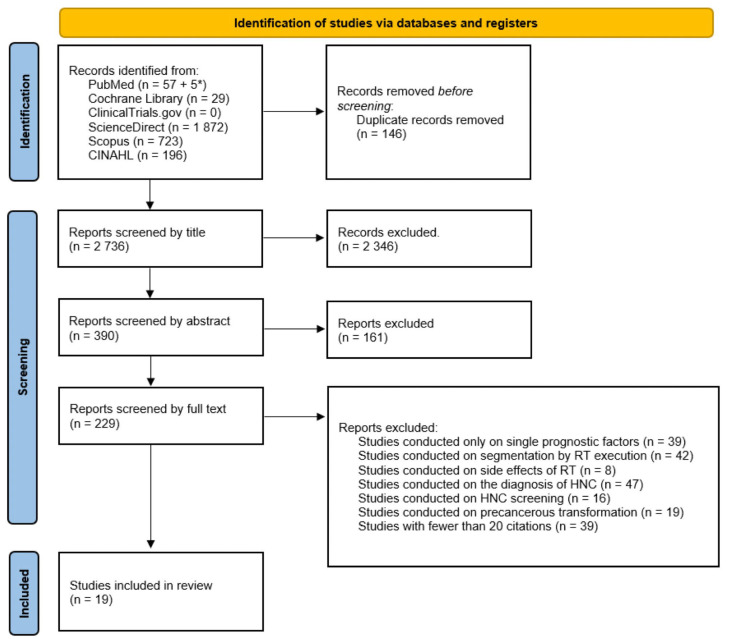
PRISMA flowchart of the systematic review process. * The first number indicates the results obtained via the keywords, while the second number refers to results identified via MeSH.

After a primary search, the articles were imported into EndNote21 (Clarivate, Analytics, Philadelphia, PA, USA), and all saved articles were screened by two independent investigators (L.M. and A.T.) through an evaluation of the title and abstract, as reported in the PRISMA flowchart (Figure 1). In case of doubts or disagreements between the two investigators, a third independent investigator (M.R.) was involved.

### 2.2. Inclusion and Exclusion Criteria

The two independent investigators (L.M.) and (A.T.) applied the following inclusion and exclusion criteria by first evaluating the title of the collected studies, then the abstract, and finally, for the remaining studies, by reading the full text. Non-randomized and randomized clinical trials, cohort studies, original articles, and research articles published from 2013 to 2023 in English only and performed on humans were included. From the results obtained from the database search, all studies dealing with artificial intelligence applied for head and neck cancer diagnosis and screening, detection and prediction of transformation of potentially malignant lesions, and segmentation used for radiotherapy planning were not considered. In addition, review articles and meta-analyses were not considered. Studies for which an abstract was not available were also excluded. Studies conducted on single prognostic factors were removed, except for those that evaluated the application of artificial intelligence for the assessment and prediction of lymph node status. Studies with fewer than 20 citations were removed.

To summarize the studies included in this review, they were grouped into two categories based on the type of topic (Table 2):

1. Prognostic models (n = 11).
2. Diagnosis and prediction of lymph node status (n = 8).

The first group included studies evaluating the efficacy of prognostic models based on artificial intelligence algorithms and studies demonstrating how these prognostic models can have a significant impact on patients’ treatment strategy, while the second group included studies evaluating the efficacy of artificial intelligence algorithms in assessing lymph node metastasis, diagnosing and predicting extracapsular lymph node extent, and predicting lymph node metastasis.

### 2.3. Data Collection

From the selected studies, the following data were extracted: study topic, study objective, endpoints examined, number of patients examined, treatment of patients examined, type of tumor examined (oral squamous cell carcinoma, oropharyngeal carcinoma, hypopharyngeal carcinoma, nasopharyngeal carcinoma, or laryngeal carcinoma), data analyzed to create the algorithm (clinical, pathological, imaging, or genetic/molecular data), the algorithm used (ML or DL), comparison (to other artificial intelligence algorithms or prognostic models based on clinical/histologic data such as TNM staging), and the summary of results obtained.

Data were manually extracted by the two independent researchers (L.M. and A.T.) and collected in a Microsoft Excel spreadsheet. This information was then displayed in Table 3; Table 4 in the Section 3.

### 2.4. Bias Assessment

**Figure 2:**
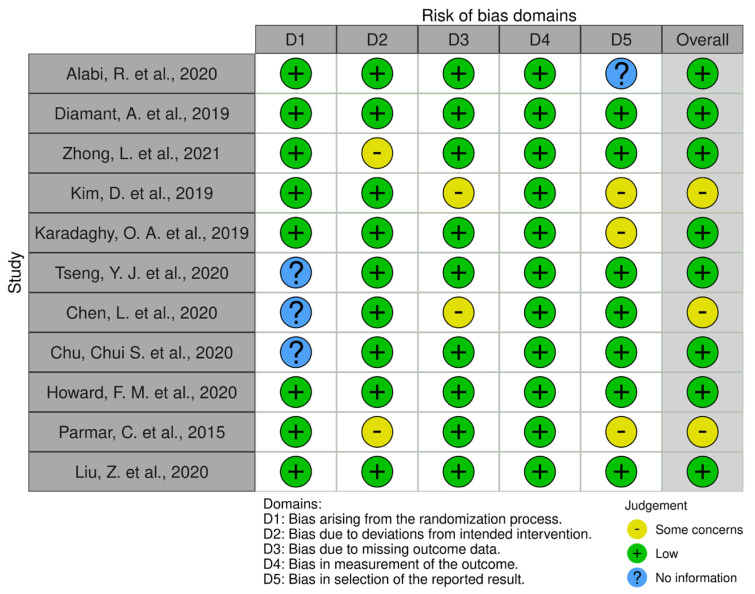
Robvis tool for assessing the risk of bias in studies concerning prognostic models.

**Figure 3:**
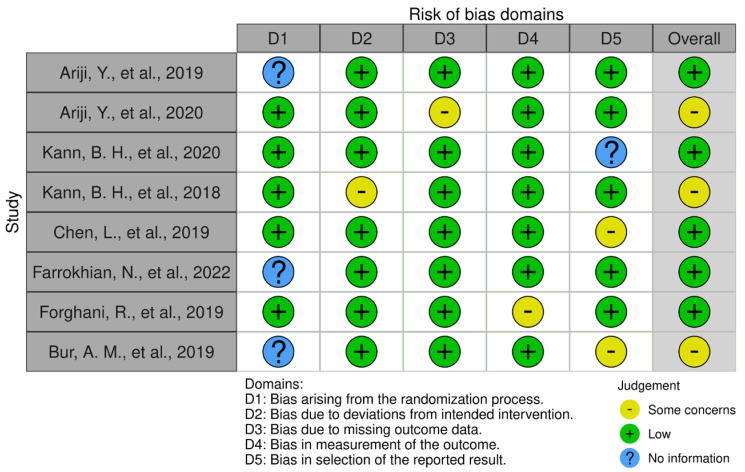
Robvis tool for assessing the risk of bias in studies concerning evaluation and prediction of lymph node status.

The risk of bias was assessed by the two independent investigators (L.M.) and (A.T.) using the Robvis tool [14]. Five types of bias were assessed: bias arising from the randomization process, bias due to deviations from the intended interventions, bias due to missing outcome data, bias in outcome measurement, and bias in reported outcome selection (Figure 2 and Figure 3).

## 3. Results

Figure 2 shows the PRISMA flowchart describing the study selection process. Using a combination of keywords, the investigators retrieved 2882 studies. All articles were imported into EndNote. After identifying duplicates, 146 studies were removed. The remaining 2736 were screened by title, with the removal of 2346 papers. Subsequent screening by abstract led to the exclusion of an additional 161 reports. The full texts of 229 studies were read and, following the application of the inclusion and exclusion criteria, 19 studies were included in this systematic review.

Of the 19 selected studies, we assessed the risk of bias using the Robvis tool [14]; 11 articles concerning prognostic models are shown in Figure 2, and 8 articles concerning lymph node status assessment are shown in Figure 3.

The included studies were grouped into two groups based on the type of topic addressed (Table 2): “prognostic models” and “evaluation and prediction of lymph node status”. The following data were collected for prognostic models (as summarized in Table 3) and for lymph node status assessment (as summarized in Table 4): study topic, study objective, endpoints examined, number of patients examined, treatment of patients examined, type of tumor examined (oral squamous cell carcinoma, oropharyngeal carcinoma, hypopharyngeal carcinoma, nasopharyngeal carcinoma, or laryngeal carcinoma), data analyzed to create the algorithm (clinical, pathological, imaging, or genetic/molecular data), algorithm used (machine learning or deep learning), comparison (to other artificial intelligence algorithms or prognostic models based on clinical/histologic data such as TNM staging), and summary of results obtained.

### 3.1. Prognostic Models (Table 3)

Several studies demonstrated that they succeeded in developing prognostic models capable of assessing parameters, such as overall survival (OS) and disease-free survival (DFS), by processing clinical, imaging, histopathological, and/or genomic data.

#### 3.1.1. Endpoint

Analyzing the data collected and summarized in Table 3, the following endpoints were evaluated from these studies: recurrence-free survival, distant metastasis-free survival, loco-regional failure, overall survival, tumor-related death, and disease-free survival. This demonstrates the broad ability of these algorithms to evaluate multiple prognostic endpoints.

#### 3.1.2. Data Analyzed

The data processed by the different artificial intelligence algorithms to obtain these prognostic results are also varied: about 73% of the studies analyze clinical data, 64% clinical/pathological data (age, sex, grading, depth of invasion, perineural invasion, lymph/vascular invasion, tumor budding, bone marrow invasion, persistence of tumor at resection margin, extranodal extension, tumor site), 36% analyze treatments already performed on the patient (radiotherapy, chemotherapy, adjuvant CT-RT, concomitant CT-RT, cervical dissection, surgical resection of primary tumor), 45% analyze imaging data with radiomic processing and patterns (CT images, CT with contrast medium, MRI, PET, PET-TC), 18% analyze socio-demographic data, and 9% analyze genetic data. All this information is not simply processed as individual data but is integrated because of the ability of artificial intelligence algorithms to do so. In fact, as the study conducted by Tseng, Y.J. et al. (2020) [15] shows, the integration of genetic data together with clinical/pathological data goes a long way toward improving the performance of the prognostic model in assessing recurrence-free survival (endpoint examined by this study).

#### 3.1.3. Types of Head and Neck Tumors Studied

The tumors analyzed are oral squamous cell carcinoma (addressed in 64% of the selected studies), oropharyngeal carcinoma (in 36%), hypopharyngeal carcinoma (in 27%), nasopharyngeal carcinoma (in 18%), and laryngeal carcinoma (in 45%).

#### 3.1.4. Artificial Intelligence Algorithms

The algorithms employed in the different studies are various, from ML models to DL models to convolutional neural networks. These models once developed are trained with data from the training cohort (training set) and then validated (analyzing the processing by these models of data from the validation cohort (testing set). They are then compared either to other artificial intelligence algorithms or to prognostic models based on clinical/pathological data (such as TNM staging or DOI (depth of invasion)). For instance, in the Alabi study (R. et al. (2020) [16]), a first comparison is made between several different types of artificial intelligibility algorithms to assess which one has better performance and to compare the most accurate model to that obtained from the DOI (depth of invasion) study.

A total of 64% of the studies reported in Table 3 evaluate the application of ML algorithms for prognostic modeling, while 36% analyze applications of DL.

The algorithms used in the selected studies are: support vector machine (SVM), naïve Bayes (NB), boosted decision tree (BDT), decision forest (DF), convolutional neural network (CNN), random forest (RF), random survival forest (RSF), linear regression (LR), decision tree (DT), support vector machine (SVM), k-nearest neighbors (KNN), bagging (BAG), Bayesian (BY), boosting (BST), decision Tree (DT), generalized linear models (GLM), multiple adaptive regression splines (MARS), nearest neighbors, neural network (Nnet), and partial least square and principle component regression (PLSR).

#### 3.1.5. Comparison

About 27% of the studies reported in Table 3 compare artificial intelligence algorithms to prognostic models based solely on the study of staging or other clinical/histologic parameters such as DOI (depth of invasion). About 36% of the reported studies compare the performance of different types of prognostic algorithms in processing the same data, while 18% of the studies compare the same algorithm in processing different data. There are several studies in the current literature that analyze the use of certain artificial intelligence algorithms but without performing a comparison, evaluating only their performance in deriving prognostic data (about 27% of the reported studies).

**Table 3:** Studies evaluating the effectiveness of prognostic models based on artificial intelligence algorithms.

	Endpoint Analyzed	Objective of the Study	Tumor Type Examined	Data Analyzed	Algorithm Used	Results
Alabi et al., 2020 [16]	Risk of recurrences in early stage	Evaluation of 4 ML algorithms in the prediction of loco-regional recurrence risk	OTSCC	Clinical/pathological data and treatment given.	ML: 4 ML algorithms (SVM, NB, BDT, DF)	BDT proved to be the best among the 4 algorithms and was then compared to a DOI-based prognostic model. Using the DOI model, only 49.5% of the examinees were correctly identified as having recidivism, while the BDT correctly recognized 78.9% as having recidivism.
Diamant et al., 2019 [17]	Risk of distant metastasis, loco-regional failure, overall survival (OS)	Compare traditional radiomic framework to CNN algorithm for predicting treatment outcomes	OPC, HPC, NPC, and LC	CT images pre-treatment	DL: CNN	The use of a single end-to-end CNN trained de novo (without secondary automatic learning algorithms) predicts oncology outcomes better than “traditional radiomics”, i.e., an approach in which an automatic learning algorithm such as random forest is used.
Kim et al., 2019 [18]	Risk of loco-regional recurrence and tumor-related death	Comparison of CPH model, RSF and DeepSurv	OSCC	Clinical/pathological data and treatment (postoperative radiation therapy, postoperative CCRT)	DL: DeepSurv	DeepSurv (DL) shows better performance among the three models (CPH, RSF, and DeepSurv) in prognostic evaluation and derivation of the following endpoints: risk of loco-regional recurrence and tumor-related death.
Karadaghy et al., 2019 [19]	Overall survival (OS)	Develop a prediction model using ML and compare it to a prediction model created by the TNM stage	OSCC	Clinical/pathological and socio-demographic data	ML: 2-class DF architecture	ML algorithm proves to have greater accuracy and precision than TNM-based model for 5-year OS assessment.
Tseng et al., 2020 [15]	Cancer-specific survival, loco-regional recurrence-free survival, distant metastasis-free survival	Develop a risk stratification model and compare the performance of the same ML algorithm but with different data processing.	OSCC	Clinical, pathological, and genetics data	ML: EN (model based on both clinical/pathological and genetic data)	In postoperative patients treated with adjuvant CCRT, the model that also included genetic data improved the prediction of loco-regional recurrence-free survival compared with the model that did not include genetic data.
Chen et al., 2020 [20]	Overall survival (OS)	Assess the prognostic value of radiomic signature and nomogram based on CT with contrast	LC	CT with contrast enhancement, clinical/pathological data	ML: Radiomic nomogram	Radiomic nomogram manages to discriminate better than cancer staging in the training cohort and validation cohort
Chu et al., 2020 [21]	Risk of loco-regional recurrence and distant metastasis	Evaluation of ML algorithms to improve prediction of clinical outcomes	OSCC	Clinical/pathological data, cervical lymph node dissection, and/or adjuvant CT-RT regimens	ML: LR, DT, SVM, KNN	The DT model was better in identifying progressive disease by analyzing the risk of local recurrence and distant metastasis.
Parmar et al., 2015 [22]	OS	Evaluation, in terms of OS prediction and ML classification methods	OPC and LC	CT images	ML: BAG, BY, BST, DT, GLM, MARS, NN, Nnet, PLSR, RF, SVM	Through AUC, the prognostic performance of different ML methods and the feasibility of these algorithms in deriving the patient’s overall survival (OS) by processing imaging data (CT images) was evaluated.
Liu et al., 2020 [23]	OS and DFS	Assessing the prediction of OS and DFS through the use of models based on radiomic signatures	OPC, LC, HPC, and OSCC	18F-PET/CT pre- and posttreatment	ML: Model with clinicopathological + radiomic data	Combining clinicopathologic and radiologic data substantially improves the prediction of OS and DFS compared with models that analyze only clinicopathologic data.
Zhong et al., 2021 [24]	Predicting the prognosis of patients with NPC with different optimal treatment regimens with DFS as the primary endpoint	Development of a DL-based model for treatment decision making by comparing CPTDN to 3 clinical models (the first based on all patients, the second only on those who received CCRT, and the third on those who received ICT + CCRT.	NPC	MR images pretreatment and clinical factors	CPTDN based on a shared backbone Nnet and two subnetworks to simultaneously predict prognosis and treatment response	Excellent prognostic ability for DFS in both the group receiving CCRT and the group receiving ICT + CCRT. Based on the prognostic difference between the two types of treatments, patients were divided into two groups: preferential ICT and preferential CCRT. In the first group, patients who received ICT + CCRT had better DFS than those who received CCRT. In the second group, however, the opposite trend occurred.
Howard et al., 2020 [25]	OS associated with treatment according to model recommendations	Identify patients with intermediate risk head and neck cancer who would benefit from adjuvant chemotherapy	OSCC, OPC, HPC, and LC	Demographic, clinical/pathological data, treatment (chemotherapy performance, radiation dose)	DL: DS, NMLR, and SF models	The 3 DL models recommended CT-RT in 44–52% of the cases examined. It was shown to have the potential for stratifying patients and selecting those who would need trimodal therapy (and excluding those who would not need this treatment approach, avoiding possible complications related to chemotherapy and RT).

Shown in blue are the two studies that represent an example of how these prognostic models may impact the choice of therapeutic strategy. Legends: AUC—area under the curve; BAG—bagging; BY—Bayesian; BDT—boosted decision tree; BST—boosting; CCRT—concurrent chemoradiotherapy; CNN: convolutional neural networks; CPH: Cox proportional hazards model; CPTDN: combined prognosis and treatment decision nomogram; CT: computed tomography; CT-RT—chemotherapy–radiotherapy; DOI—depth of invasion; DL—deep learning; DF—decision forest; DFS—disease free survival; DS—DeepSurv; DT—decision tree; EN—elastic net; GLM—generalized linear models; HPC—hypopharyngeal cancer; ICT—induction chemotherapy; KNN—k-nearest neighbors; LC—laryngeal cancer; LR—linear regression; ML: machine learning; MARS: Multiple adaptive regression splines; NB: Naïve Bayes; NMLR: neural multitasking logistic regression; NN: Nearest neighbors; Nnet—neural networks; NPC—nasopharyngeal carcinoma; OPC—oropharyngeal cancer; OS—overall survival; OSCC—oral squamous cell carcinoma; OTSCC—oral tongue squamous cell carcinoma; PET—positron emission tomography; PLSR—partial least square and principle component regression; RF—random forest; RSF—random survival forest; RT—radiotherapy; MR—magnetic resonance; SF—survival forest; SVM—support vector machine.

#### 3.1.6. Evaluation of Prognostic Endpoint

The application of ML and DL algorithms have proven to be very useful in evaluating different types of endpoints, including recurrence-free survival, distant metastasis-free survival, loco-regional failure, overall survival, tumor-related death, and disease-free survival.

The study conducted by Alabi et al. (2020) [16] compared the performance of four different types of ML algorithms (support vector machine, naïve Bayes, boosted decision tree, and decision forest) in deriving the risk of recurrence in patients with oral tongue squamous cell carcinoma by processing clinical/pathological data. The algorithm that proved best among the four, namely the boosted decision tree (BDT), was then compared to a prognostic model based solely on DOI (depth of invasion). This study showed how the DOI model correctly identified only 49.5 percent of patients with recurrence, while the ML model (the BDT) recognized 78.9 percent, demonstrating greater accuracy. Another study demonstrated how artificial intelligence algorithms were more effective in assessing the overall survival of five patients with oral squamous cell carcinoma (OSCC), namely the study conducted by Karadaghy et al. (2019) [19]. This study compared an ML algorithm to a prognostic model obtained through TNM staging, demonstrating how the former had greater accuracy and precision than the latter in calculating overall survival.

As mentioned earlier, studies comparing the same type of diagnostic algorithm but with different data processing were included. The study conducted by Liu et al. (2020) [23] showed that processing by the same ML algorithm of both clinical/pathological data together with radiomic data obtained from PET/CT scans was better in predicting overall survival (OS) and disease-free survival (DFS) than processing clinical data alone in a patient with oropharyngeal, laryngeal, hypopharyngeal, and oral cavity cancer. The study conducted by Tseng et al. (2020) [15] showed that processing both clinical data and genetic data, again performed by the same ML algorithm, was better in predicting cancer-specific survival, loco-regional recurrence-free survival, and distant metastasis-free survival than processing only clinical/pathological data in patients with oral squamous cell carcinoma.

In contrast, the study conducted by Diamant et al. (2019) [17], which examined patients with oropharyngeal, hypopharyngeal, nasopharyngeal, and laryngeal cancer, showed how the DL algorithm (the convolutional neural network) performed better than the ML (random forest) algorithm in calculating the risk of distant metastasis (DM), loco-regional failure (LRF), and overall survival (OS) by analysis of CT images performed during presurgical treatment.

### 3.2. Diagnosis and Prediction of Lymph Node Status (Table 4)

Knowing the lymph node status is crucial in the management of a patient with a head/neck tumor. The following studies have been divided into three parts:

1. Studies on the “Assessment of cervical lymph node metastasis”;
2. Studies on the “Diagnosis and prediction of ENE (extranodal extension)”;
3. Studies on “Prediction of lymph node metastasis”.

#### 3.2.1. Topics

The topics of these studies are to develop models based on artificial intelligence in order to perform more precise diagnoses and evaluation of lymph node metastasis, to diagnose and predict the occurrence of extranodal extension (ENE), and to predict the occurrence of lymph node metastasis.

#### 3.2.2. Data Analyzed

The data analyzed for the evaluation of these prognostic models are mostly radiomic in nature: 75% of the selected studies present imaging data for the creation and validation of machine learning and deep learning algorithms, while the remaining 25% exploit clinical/pathological data. Particularly, of that 75%, data come from CT scans (66%), PET-CT (17%), and DECT dual-energy CT (17%).

#### 3.2.3. Type of Head and Neck Tumors Studied

The tumors analyzed are oral squamous cell carcinoma (addressed in 87% of the selected studies), oropharyngeal carcinoma (50%), hypopharyngeal carcinoma (37%), nasopharyngeal carcinoma (25%), and laryngeal carcinoma (50%).

#### 3.2.4. Comparison

A total of 37.5% of the studies reported in Table 4 compare the performance of artificial intelligence algorithms to the analytical ability of professional radiologists (in studies in which the data to be analyzed are CT images), 37.5% of the studies compare these algorithms to models based on the study of clinical/pathological factors (such as DOI), 12.5% of the studies compare different types of algorithms to the same types of data processed, and another 12.5% of the studies reported compare the performance of the same algorithms but with different data processing.

**Table 4:** Studies evaluating assessment and prediction of lymph node status.

	Topics	Objective of the Study	Tumor Type Examined	Data Analyzed	Algorithm Used	Results
Ariji et al., 2019 [26]	Assessment of cervical lymph node metastasis	Evaluation of DL performance for diagnosis of lymph node metastasis	OSCC	CT images	DL	The results obtained by DL models are similar to those obtained by experienced radiologists in terms of accuracy, sensitivity, specificity, and positive and negative predictive value. This indicates the reliability of these algorithms in the diagnosis of lymph node metastasis and their potential use as an aid tool for radiologists.
Ariji et al., 2020 [27]	Diagnosis and prediction of ENE	Clarifying the diagnostic performance of CT in ENE by applying DL algorithms	OSCC	CT images	DL	The diagnostic performance of the DL is superior to that obtained by radiologists.
Kann et al., 2020 [28]	Diagnosis and prediction of ENE	Evaluation of the application of DL algorithms in the pretreatment identification of ENEs	OPC, LC, HPC, NPC, OSCC	Contrast-enhanced CT scans	DL	The DL algorithm achieved higher AUC values than those obtained by the two radiologists. Implementation of these DL algorithms in a radiologist’s work would provide increased AUC and sensitivity.
Kann et al., 2018 [29]	Diagnosis and prediction of ENE	Evaluation of the application of DL algorithms in the prediction of lymph node metastasis and ENE	OPC, LC, HPC, NPC, OSCC	CT images	DL	In the testing set, DL predicted ENE and lymph node metastasis with an AUC of 0.91, while the logistic regression model (based on clinical risk factors and lymph node ROI diameter) obtained an AUC of 0.81.
Chen et al., 2019 [30]	Prediction of lymph node metastasis	Evaluation of an automatic prediction model for lymph node metastasis	OPC and LC	CT-PET images pretreatment	“Hybrid” predictive model based on radiomics and DL strategies (fusion of MaO-radiomics and 3D-CNN)	Hybrid model achieved higher accuracy than XmasNet and radiomics models.
Farrokhian et al., 2022 [31]	Prediction of lymph node metastasis	Evaluation of an ML model for prediction of occult early-stage lymph node metastasis	OSCC	Clinical/pathological data (obtained after surgical resection of the primary tumor)	ML	The ML predictive model outperformed the DOI-based model in terms of AUC, sensitivity, specificity, and positive and negative predictive value.
Forghani et al., 2019 [32]	Prediction of lymph node metastasis	Development and evaluation of risk stratification model by dual-energy texture analysis (DECT) by ML to predict lymphadenopathy	OPC, HPC, LC, OSCC	Dual-energy by texture analysis (DECT)	ML (RF patterns)	The application of an ML algorithm in the analysis of multi-energy DECT textures has been shown to be superior in predicting lymph node metastasis compared with models based solely on single-energy CT.
Bur et al., 2019 [33]	Prediction of lymph node metastasis	Development and evaluation of an ML algorithm to predict lymph node metastasis	OSCC	Clinical/pathological data	ML (DF algorithm)	The decision forest algorithm outperformed the performance achieved by the DOI model in the external test.

Studies dealing with the diagnosis of lymph node metastasis are shown in yellow, those dealing with the assessment and the prediction of extranodal extension (ENE) are shown in blue, and those dealing with the prediction of lymph node metastasis are shown in green. Legends: AUC—area under the curve; CT—computed tomography; CNN—convolutional neural net; DECT—dual-energy computed tomography; DF—decision forest; DL—depth learning; DOI—deep of invasion; ENE—extranodal extension; HPC—hypopharyngeal cancer; LC—laryngeal cancer; ML—machine learning; NPC—nasopharyngeal cancer; OPC—oropharyngeal cancer; OSCC—oral squamous cell carcinoma; RF—random forest.

#### 3.2.5. Lymph Node Status Assessment

Artificial intelligence algorithms have proven to be especially useful in diagnosing and predicting lymph node metastasis and predicting extranodal extension (a very important prognostic factor that severely impacts a patient’s prognosis). The study conducted by Ariji et al. (2019) [26] investigated the ability of DL to diagnose lymph node metastasis in patients with oral squamous cell carcinoma through CT image processing. The results obtained were then compared to those of two experienced radiologists. It was shown that the results obtained by the DL models were similar to those obtained by the two radiologists in terms of accuracy, sensitivity, and specificity, emphasizing how these algorithms can be useful tools to be used in the work of radiologists.

On the subject of extranodal extension, the study conducted by Ariji et al. (2020) [27] instead examined the ability of DL algorithms to diagnose the presence of ENE in patients with oral squamous cell carcinoma. The performance of these algorithms was shown to be superior to that of three radiologists who were tasked with reviewing the same CT images. In contrast, the study conducted by Kann et al. (2018) [29] focused on evaluating the ability of DL algorithms in predicting ENE in patients with oropharyngeal, laryngeal, hypopharyngeal, nasopharyngeal, and oral cavity cancer. The DL model was compared to a regression model of clinical risk factors and diagnostic controls performed by radiologists, showing that the application of the DL algorithm achieved superior performance in predicting both ENE and lymph node metastasis.

Regarding the prediction of lymph node metastasis, another study also demonstrated interesting results. The study by Farrokhian et al. (2022) [31] compared the performance of an ML algorithm to that of a model that relied solely on DOI (depth of invasion) assessment, showing how the predictive ML model was better in terms of AUC (area under the curve), sensitivity, and specificity.

## 4. Discussion

As stated in the introduction, the purpose of this paper is to provide an overview of the potential performance of artificial intelligence as applied in the prognostic evaluation of head and neck cancer patients, particularly in prognostic modeling, clinical endpoint assessment, and lymph node status assessment.

When the clinician assesses the prognosis of the cancer patient, several prognostic factors are assessed through the study of the clinical, imaging, and histologic report and the possible presence of certain molecular alterations that, in addition to having prognostic significance, may add predictive value to the use of certain treatments. We understand how precisely knowing the patient’s prognosis allows for the best management of the disease with proper therapy and follow-up.

The application of artificial intelligence algorithms is proving useful for this purpose because of their ability to process information in a way that the human mind alone cannot. Studies demonstrating the validity of ML for predicting treatment outcomes in cancers such as prostate and breast cancer have been published for some time now [34].

There are recent studies demonstrating how the application of artificial intelligence algorithms can be a useful tool for outcome prediction in patients with head and neck cancer, such as the study conducted by Chinnery et al. (2021) [5] that demonstrated how the use of these algorithms can be applied in prognostic evaluation through the analysis of imaging data, the study conducted by Adeoye et al. (2021) [35] that demonstrated how these tools have excellent accuracy in predicting both lymph node metastasis and prognosis in the patient with oral cavity cancer, or the study conducted by Zhang et al. (2023) [36] that showed how radiomics can be a means to the clinician’s advantage in assessing clinical endpoints.

From the studies we have collected in our review, it can be understood how ML and DL algorithms provide excellent performance in assessing different types of prognostic endpoints (such as, for example, overall survival) and in predicting posttreatment outcomes, demonstrating how they can be an essential tool to better personalize therapy. The application of these algorithms has demonstrated greater accuracy both in terms of AUC (area under the curve) and in terms of specificity and sensitivity than models commonly used in clinical practice for prognostic evaluation, such as prognostic models based on TNM staging or DOI (depth of invasion). Moreover, by being able to process multiple types of information (clinical, radiological, histological, and molecular data), together these tools have demonstrated a greater ability to stratify patients according to their prognosis by even being able to identify which subgroups of patients with the same tumor and stage would benefit from specific treatments and which would not. In this regard, the study conducted by Howard et al. (2020) [25] (that we include in our review) demonstrated how different DL algorithms (DeepSurv, neural multitasking logistic regression, and survival forest) were able, through the analysis of demographics and clinical/pathological data and according to the type of treatment received, to stratify patients with early-stage head and neck cancer into different subgroups, identifying those who would receive a benefit from adding adjuvant chemotherapy to surgical treatment. This has a major impact on the lives of patients, as it means more aggressive treatments against early-stage cancer in those who have a higher risk of recurrence and metastasis, while sparing those who would not benefit from such treatments from the side effects of chemotherapy and radiotherapy (mucositis, osteonecrosis, dermatitis, dysphagia, and many others) [37,38,39].

Studies conducted on lymph node metastasis prediction also prove to be useful for patient stratification and therapy personalization, such as the study conducted by Farrokhian et al. (2022) [31] that examined the application of ML in lymph node metastasis prediction and demonstrated how it is superior to the DOI (depth of invasion)-based prognostic model, succeeding in selecting which patients with early-stage head and neck cancer would benefit from cervical lymph node dissection. Thus, artificial intelligence algorithms prove to be much more accurate in predicting lymph node metastasis and extranodal extension (ENE) than assessments conducted by DOI (depth of invasion)-based models. For the diagnosis of lymph node metastasis, through the study of radiologic images, we have observed how these algorithms perform the same if not in some cases even better than those of experienced radiologists. This shows, in our opinion, how these algorithms can be considered reliable and how they can be used as an auxiliary tool for the clinician in tumor assessment.

However, we must emphasize some aspects that we consider limiting. Although these algorithms show excellent performance in prognostic evaluation, further studies are needed to have a significant impact on clinical practice, especially through the implementation of standardized protocols. Ther are numerous studies addressing the issue of head and neck cancer prognosis, but not all of them analyze the same algorithms and the same types of data (clinical, radiological, histological, and molecular), not to mention the type of treatment received by the patient. Moreover, the fact that there are studies analyzing the same types of prognostic factors for different types of head and neck cancer that could represent a risk, they would neglect the study of prognostic factors that are peculiar only to certain types of cancer, such as HPV positivity. In oropharyngeal cancer, the presence of HPV has a recognized prognostic role, while, for oral squamous cell carcinoma, they recognize this role during prognosis [40,41]. It must also be borne in mind that certain types of data, such as molecular data, cannot be obtained in all hospitals, as not all centers have the required facilities and laboratories.

## 5. Conclusions

Although more studies and standardized protocols are needed for them to have a significant impact on clinical practice, artificial intelligence algorithms demonstrate excellent performances in predicting outcomes after treatment, evaluating clinical endpoints, and predicting metastasis and recurrence in head and neck cancer patients. These algorithms exhibit better accuracy than commonly used prognostic models such as those that rely on TNM staging or DOI (depth of invasion). The application of ML and DL algorithms in prognostic evaluation has also shown how it is possible to stratify cancer patients with the same tumor and at the same stage into multiple subgroups, identifying which patients would benefit from more aggressive treatments toward the tumor (such as, for example, the trimodal approach of surgery, chemotherapy, and radiotherapy) and who would not, with the aim of avoiding the patient side effects that would result from such an approach. AI is showing great promise. However, prospective clinical trials comparing AI to standard prognostic algorithms are required to evaluate AI as a tool for disease management. We believe that the application of artificial intelligence in the management of oncology patients can play an important role in the medicine of the future.

## Figures and Tables

**Table 1:** PICOS framework.

Participant	Patients with head and neck cancer that have already been diagnosed.
Interventions	Evaluation of prognostic factors using artificial intelligence algorithms.
Comparators	Comparison to other artificial intelligence algorithms or the same algorithm but with different types of data processing or prognostic models based on clinical pathological data (e.g., TNM staging).
Outcomes	Recurrence-free survival, distant metastasis-free survival, loco-regional failure, overall survival, tumor-related death, and disease-free survival.
Study design	Clinical trials, randomized clinical trials, cohort studies, research articles, and original articles

**Table 2:** Breakdown of selected articles by topic.

Main Groups	Topics
Prognostic models (n = 11)	Creation of prognostic models (n = 9); b. Example of prognostic models with the ability to guide therapeutic choice (n = 2).
2. Diagnosis and prediction of lymph node status (n = 8)	Diagnosis of lymph node metastasis (n = 1); b. Diagnosis and prediction of extracapsular lymph node extension (n = 3); c. Prediction of lymph node metastasis (n = 4).

## Supplementary Materials

The following supporting information can be downloaded at: https://www.mdpi.com/article/10.3390/jpm13121626/s1.

## References

[1] Vigneswaran N., Williams M.D. (2014). Epidemiologic Trends in Head and Neck Cancer and Aids in Diagnosis. Oral Maxillofac. Surg. Clin. N. Am..

[2] Bray F., Ferlay J., Soerjomataram I., Siegel R.L., Torre L.A., Jemal A. (2018). Global Cancer Statistics 2018: GLOBOCAN Estimates of Incidence and Mortality Worldwide for 36 Cancers in 185 Countries. CA Cancer J. Clin..

[3] Chen M.M., Terzic A., Becker A.S., Johnson J.M., Wu C.C., Wintermark M., Wald C., Wu J. (2022). Artificial Intelligence in Oncologic Imaging. Eur. J. Radiol. Open.

[4] Abdel Razek A.A.K., Khaled R., Helmy E., Naglah A., AbdelKhalek A., El-Baz A. (2022). Artificial Intelligence and Deep Learning of Head and Neck Cancer. Magn. Reson. Imaging Clin. N. Am..

[5] Chinnery T., Arifin A., Tay K.Y., Leung A., Nichols A.C., Palma D.A., Mattonen S.A., Lang P. (2021). Utilizing Artificial Intelligence for Head and Neck Cancer Outcomes Prediction From Imaging. Can. Assoc. Radiol. J..

[6] Mahmood H., Shaban M., Indave B.I., Santos-Silva A.R., Rajpoot N., Khurram S.A. (2020). Use of Artificial Intelligence in Diagnosis of Head and Neck Precancerous and Cancerous Lesions: A Systematic Review. Oral Oncol..

[7] Resteghini C., Trama A., Borgonovi E., Hosni H., Corrao G., Orlandi E., Calareso G., De Cecco L., Piazza C., Mainardi L. (2018). Big Data in Head and Neck Cancer. Curr. Treat. Options Oncol..

[8] Mäkitie A.A., Alabi R.O., Ng S.P., Takes R.P., Robbins K.T., Ronen O., Shaha A.R., Bradley P.J., Saba N.F., Nuyts S. (2023). Artificial Intelligence in Head and Neck Cancer: A Systematic Review of Systematic Reviews. Adv. Ther..

[9] Gharavi S.M.H., Faghihimehr A. (2022). Clinical Application of Artificial Intelligence in PET Imaging of Head and Neck Cancer. PET Clin..

[10] Peng Z., Wang Y., Wang Y., Jiang S., Fan R., Zhang H., Jiang W. (2021). Application of Radiomics and Machine Learning in Head and Neck Cancers. Int. J. Biol. Sci..

[11] Chow L.Q.M. (2020). Head and Neck Cancer. N. Engl. J. Med..

[12] Karatzanis A.D., Koudounarakis E., Papadakis I., Velegrakis G. (2012). Molecular Pathways of Lymphangiogenesis and Lymph Node Metastasis in Head and Neck Cancer. Eur. Arch. Otorhinolaryngol..

[13] Page M.J., McKenzie J.E., Bossuyt P.M., Boutron I., Hoffmann T.C., Mulrow C.D., Shamseer L., Tetzlaff J.M., Akl E.A., Brennan S.E. (2021). The PRISMA 2020 Statement: An Updated Guideline for Reporting Systematic Reviews. BMJ.

[14] McGuinness L.A., Higgins J.P.T. (2021). Risk-of-bias VISualization (Robvis): An R Package and Shiny Web App for Visualizing Risk-of-bias Assessments. Res. Synth. Methods.

[15] Tseng Y.-J., Wang H.-Y., Lin T.-W., Lu J.-J., Hsieh C.-H., Liao C.-T. (2020). Development of a Machine Learning Model for Survival Risk Stratification of Patients with Advanced Oral Cancer. JAMA Netw. Open.

[16] Alabi R.O., Elmusrati M., Sawazaki-Calone I., Kowalski L.P., Haglund C., Coletta R.D., Mäkitie A.A., Salo T., Almangush A., Leivo I. (2020). Comparison of Supervised Machine Learning Classification Techniques in Prediction of Locoregional Recurrences in Early Oral Tongue Cancer. Int. J. Med. Inform..

[17] Diamant A., Chatterjee A., Vallières M., Shenouda G., Seuntjens J. (2019). Deep Learning in Head & Neck Cancer Outcome Prediction. Sci. Rep..

[18] Kim D.W., Lee S., Kwon S., Nam W., Cha I.-H., Kim H.J. (2019). Deep Learning-Based Survival Prediction of Oral Cancer Patients. Sci. Rep..

[19] Karadaghy O.A., Shew M., New J., Bur A.M. (2019). Development and Assessment of a Machine Learning Model to Help Predict Survival Among Patients with Oral Squamous Cell Carcinoma. JAMA Otolaryngol. Head Neck Surg..

[20] Chen L., Wang H., Zeng H., Zhang Y., Ma X. (2020). Evaluation of CT-Based Radiomics Signature and Nomogram as Prognostic Markers in Patients with Laryngeal Squamous Cell Carcinoma. Cancer Imaging.

[21] Chu C.S., Lee N.P., Adeoye J., Thomson P., Choi S. (2020). Machine Learning and Treatment Outcome Prediction for Oral Cancer. J. Oral. Pathol. Med..

[22] Parmar C., Grossmann P., Rietveld D., Rietbergen M.M., Lambin P., Aerts H.J.W.L. (2015). Radiomic Machine-Learning Classifiers for Prognostic Biomarkers of Head and Neck Cancer. Front. Oncol..

[23] Liu Z., Cao Y., Diao W., Cheng Y., Jia Z., Peng X. (2020). Radiomics-Based Prediction of Survival in Patients with Head and Neck Squamous Cell Carcinoma Based on Pre- and Post-Treatment 18F-PET/CT. Aging.

[24] Zhong L., Dong D., Fang X., Zhang F., Zhang N., Zhang L., Fang M., Jiang W., Liang S., Li C. (2021). A Deep Learning-Based Radiomic Nomogram for Prognosis and Treatment Decision in Advanced Nasopharyngeal Carcinoma: A Multicentre Study. EBioMedicine.

[25] Howard F.M., Kochanny S., Koshy M., Spiotto M., Pearson A.T. (2020). Machine Learning—Guided Adjuvant Treatment of Head and Neck Cancer. JAMA Netw. Open.

[26] Ariji Y., Fukuda M., Kise Y., Nozawa M., Yanashita Y., Fujita H., Katsumata A., Ariji E. (2019). Contrast-Enhanced Computed Tomography Image Assessment of Cervical Lymph Node Metastasis in Patients with Oral Cancer by Using a Deep Learning System of Artificial Intelligence. Oral Surg. Oral Med. Oral Pathol. Oral Radiol..

[27] Ariji Y., Sugita Y., Nagao T., Nakayama A., Fukuda M., Kise Y., Nozawa M., Nishiyama M., Katumata A., Ariji E. (2020). CT Evaluation of Extranodal Extension of Cervical Lymph Node Metastases in Patients with Oral Squamous Cell Carcinoma Using Deep Learning Classification. Oral Radiol..

[28] Kann B.H., Hicks D.F., Payabvash S., Mahajan A., Du J., Gupta V., Park H.S., Yu J.B., Yarbrough W.G., Burtness B.A. (2020). Multi-Institutional Validation of Deep Learning for Pretreatment Identification of Extranodal Extension in Head and Neck Squamous Cell Carcinoma. J. Clin. Oncol..

[29] Kann B.H., Aneja S., Loganadane G.V., Kelly J.R., Smith S.M., Decker R.H., Yu J.B., Park H.S., Yarbrough W.G., Malhotra A. (2018). Pretreatment Identification of Head and Neck Cancer Nodal Metastasis and Extranodal Extension Using Deep Learning Neural Networks. Sci. Rep..

[30] Chen L., Zhou Z., Sher D., Zhang Q., Shah J., Pham N.-L., Jiang S., Wang J. (2019). Combining Many-Objective Radiomics and 3D Convolutional Neural Network through Evidential Reasoning to Predict Lymph Node Metastasis in Head and Neck Cancer. Phys. Med. Biol..

[31] Farrokhian N., Holcomb A.J., Dimon E., Karadaghy O., Ward C., Whiteford E., Tolan C., Hanly E.K., Buchakjian M.R., Harding B. (2022). Development and Validation of Machine Learning Models for Predicting Occult Nodal Metastasis in Early-Stage Oral Cavity Squamous Cell Carcinoma. JAMA Netw. Open.

[32] Forghani R., Chatterjee A., Reinhold C., Pérez-Lara A., Romero-Sanchez G., Ueno Y., Bayat M., Alexander J.W.M., Kadi L., Chankowsky J. (2019). Head and Neck Squamous Cell Carcinoma: Prediction of Cervical Lymph Node Metastasis by Dual-Energy CT Texture Analysis with Machine Learning. Eur. Radiol..

[33] Bur A.M., Holcomb A., Goodwin S., Woodroof J., Karadaghy O., Shnayder Y., Kakarala K., Brant J., Shew M. (2019). Machine Learning to Predict Occult Nodal Metastasis in Early Oral Squamous Cell Carcinoma. Oral Oncol..

[34] Kourou K., Exarchos T.P., Exarchos K.P., Karamouzis M.V., Fotiadis D.I. (2015). Machine Learning Applications in Cancer Prognosis and Prediction. Comput. Struct. Biotechnol. J..

[35] Adeoye J., Tan J.Y., Choi S.-W., Thomson P. (2021). Prediction Models Applying Machine Learning to Oral Cavity Cancer Outcomes: A Systematic Review. Int. J. Med. Inform..

[36] Zhang Y.-P., Zhang X.-Y., Cheng Y.-T., Li B., Teng X.-Z., Zhang J., Lam S., Zhou T., Ma Z.-R., Sheng J.-B. (2023). Artificial Intelligence-Driven Radiomics Study in Cancer: The Role of Feature Engineering and Modeling. Mil. Med. Res..

[37] Alfouzan A.F. (2021). Radiation Therapy in Head and Neck Cancer. Saudi Med. J..

[38] Gau M., Karabajakian A., Reverdy T., Neidhardt E.-M., Fayette J. (2019). Induction Chemotherapy in Head and Neck Cancers: Results and Controversies. Oral Oncol..

[39] Dreno B. (1990). Mucocutaneous Side Effects of Chemotherapy. Biomed. Pharmacother..

[40] Huang S.H., O’Sullivan B. (2017). Overview of the 8th Edition TNM Classification for Head and Neck Cancer. Curr. Treat. Options Oncol..

[41] Hübbers C.U., Akgül B. (2015). HPV and Cancer of the Oral Cavity. Virulence.

